# Food Insecurity in Homeless Families in the Paris Region (France): Results from the ENFAMS Survey

**DOI:** 10.3390/ijerph15030420

**Published:** 2018-02-28

**Authors:** Judith Martin-Fernandez, Sandrine Lioret, Cécile Vuillermoz, Pierre Chauvin, Stéphanie Vandentorren

**Affiliations:** 1 INSERM, Sorbonne Université, Institut Pierre Louis d’Épidémiologie et de Santé Publique, Department of Social Epidemiology, 75012 Paris, France; judith.martin.eres@gmail.com (J.M.-F.); cecile.vuillermoz@inserm.fr (C.V.); pierre.chauvin@inserm.fr (P.C.); 2 INSERM, Université Sorbonne Paris Cité, Centre of Research in Epidemiology and Statistics, Early Life Research on Later Health (EARoH) Team, 75004 Paris, France; sandrine.lioret@inserm.fr; 3 Santé Publique France, French National Public Health Agency, 94410 Saint-Maurice, France; 4 Observatoire du Samusocial de Paris, 75012 Paris, France

**Keywords:** homeless, food insecurity, urban health

## Abstract

The number of families living in shelters in the Paris region (France) has increased by a factor of three in 10 years. In 2013, a survey was performed on homeless families in order to characterize their living conditions, their health needs, and the developmental problems in children. This probability survey was conducted in 17 languages among 801 homeless families sheltered in emergency centers for asylum-seekers, emergency housing centers, social rehabilitation centers, and social hotels in the Paris region. Among the 772 families that provided data on food security only 14.0% were with food security, whereas 43.3% were with low food security and 9.8% with very low food security (a situation where children are also affected). Stratified multivariate robust Poisson models showed that some characteristics are associated with a higher risk of food insecurity and/or of falling into very low food security, such as residential instability, single parenthood, having more than three children, depressive symptoms, housing in social hostels, and difficult access to cheap or free food locally. Given the wealth of the Paris region, resources and programs should be concentrated on improving the living situation of this vulnerable population. It needs better detection of these families, a closer social follow-up, and an increase in food aid.

## 1. Introduction

Homelessness is increasing in Europe, reaching record numbers across several member states. In France, in 2012, the number of homeless people was estimated at more than 140,000, representing a 50% increase since 2001 [1]. Recently, a report showed that being young, being a migrant, and having dependent family members exposes oneself to the risk of having housing difficulties [2] and that families are the fastest growing segment in the homeless population [2]. For example, in 2010 in the Paris region, the homeless hotline (a free emergency phone service to find vacancies in shelters, dedicated to homeless people in the streets) sheltered more families than individuals: more than 11,000 parents and children were accommodated, corresponding to a 300% increase of homeless families in 10 years [3]. In France, despite this dramatically increasing number of homeless families, little is known regarding their living conditions and associated health problems. Nevertheless, some studies have shown that homeless people are dramatically affected by food insecurity [4,5,6,7]. Food insecurity (FI) “exists whenever the availability of nutritionally adequate and safe foods or the ability to acquire acceptable food in socially acceptable ways is limited or uncertain” [8]. It is a risk factor for poor health [9,10]; this situation is particularly worrying at the more severe stage of FI when children are exposed. Several studies demonstrated that households with children experiencing food insecurity are at higher risks of birth defects [11], anemia and suboptimal nutrient intakes [12,13,14], poorer general health [15], chronic diseases [16,17], and mental distress [18]. In industrialized countries food insecurity has been particularly studied and monitored in the general population in the US and Canada [19,20,21]. In France, the study has been done more recently [22,23,24] in the aftermath of the 2008 financial crisis.

In this context and for the first time in France, the *Observatoire du SamuSocial de Paris* conducted in 2013 the ENFAMS (*Enfants et familles sans logement*) survey on homeless children and families in the Paris region [3] in order to describe their socio-demographic characteristics and evaluate their health status and needs. Based on this study, the number of sheltered families was estimated at more than 10,000 (about 35,000 people, including more than 17,000 children younger than 12 years).

The objectives of the present paper are to estimate the prevalence of FI in homeless families and children and the living conditions associated therewith, in order to better understand and prevent it. It aims to identify the characteristics of families that managed to remain food secure in this extreme situation and the characteristics of the families that are experiencing the most severe level of food insecurity.

## 2. Materials and Methods

### 2.1. Study Design and Participants

The ENFAMS survey design and sampling frame have been described elsewhere [3,25]. An eligible family was defined as comprising of at least one parent (>18 years old) with at least one child younger than 13 years, speaking one of the 17 languages available for the survey and able to provide written consent to participate. A complex sampling design was used to obtain a representative sample of homeless families [3] sheltered in different kind of facilities in the Paris region (homeless families temporarily housed by relatives or living in a slum or a camp were not included). This study chose to focus on emergency centers, social rehabilitation centers, social hostels, and centers for asylum seekers. Emergency shelters are generally characterized by group accommodations that provide only short-term, basic services (overnight accommodation with breakfast). Long-term rehabilitation centers and social hostels provide rooms in either a collective facility, self-contained flats, or hotel rooms with a wide variety of services (e.g., access to a kitchen) and stays lasting up to several months. Asylum seekers are usually sheltered in special centers with social and administrative aid during the whole processing of the application. However, since the early 2000s, there have been a shortage of beds in these centers in the Paris region, and asylum seekers are increasingly being accommodated in cheap hotels.

A three-level sampling method was used. We first made an exhaustive list of the 796 accommodation services of interest in the Paris region and selected a random sample of 251 (32%) services. Among them, 237 were contacted and 193 accepted to participate. A higher proportion of social hotels agreed to participate (89%), while long-term rehabilitation centers were less willing to do so (64%). Second, a random selection of six or seven families was performed in each selected service. One parent from each family was interviewed (in 95.4% of the cases this was ultimately the mother). Among the 1238 selected families, 980 (79%) accepted to participate (the desired sample size was 750 for the main objective of the study, which was to estimate the prevalence of anemia among mothers). The 258 families who refused to participate gave the following main reasons: not interested (23%), lack of time (14%), impossibility of getting the other parent’s approval (11%), refusal of any contact (7%), circumstances of the survey (4%), other language spoken (2%), or unknown reason (29%). Non-participant adults were younger (mean age: 33 vs. 38 years), more often men (15.3% vs. 4.6%) and more likely to have more than two children under care (31.7% vs. 23.1%) than participants. Among the 980 families who gave their consent to participate, 179 (14%) were lost during the survey (unreachable at the time of the interviewers or no longer in the service). Finally, 801 families were interviewed (one child being also randomly selected in every family).

### 2.2. Data Collection

After obtaining written consent, an interviewer administered a face-to-face questionnaire to the parent. It collected demographics (age, gender, country of birth, migration and residential trajectories, marital status, number of children), socio-economic characteristics (level of education, financial support, occupational status, professional experiences, housing history since the first episode of homelessness, number of accommodation moves since the beginning of the most recent episode of homelessness, social relationships, social support, and understanding of the French language). Food insecurity was measured using the Household Food Security Scale Measure (HFSSM), a scale created by the US Department of Agriculture (USDA) [26], the French translation of which was previously used in Québec [27] and in France [22]. The HFSSM questionnaire includes a preliminary question and 18 items (10 adult-referenced and 8 child-referenced). A single score ranging from 0 to 18 was calculated as the total number of affirmative responses, i.e., “yes” or “sometimes/often” to the 18 questions. It was divided into four categories defined by the usual thresholds: food security (FS, score < 3), marginal food security (MFS, score = 3–7), low food security (LFS, score = 8–12) and very low food security (VLFS, score > 13) [28,29]. The last and most severe category (VLFS) indicates disrupted eating patterns and reduced food intake that affect also the children in the Household (HH).

This study was approved by two ethics committees (the *Comité de protection des personnes* of the Paris region, and the national *Comité consultatif sur le traitement de l'information en matière de recherche*) and by the *Commission Nationale de l'Informatique et des Libertés*, in charge of the citizens’ data protection.

### 2.3. Outcomes

Due to the specificity of our population, we chose to focus on the two extreme situations: food security and very low food security. Two dichotomous variables were used in multivariable analyses: Food-secure (FS) vs. Food-insecure (i.e., MFS+LFS+VLFS, model 1) and VLFS vs. LFS (model 2). The second model was estimated in order to study specifically what might cause homeless families with low food security to fall into very low food security (the extreme food insecurity status where children are also affected).

### 2.4. Covariates

HH monthly income was calculated as the total HH income divided by the number of people in the consumption unit (CU) on the basis of the Organisation for Economic Co-operation and Development (OECD) scale [30]. It was used as a continuous numerical variable (expressed in fifties of €/CU increments), then as a stratification categorical variable (due to interactions discussed below). For this latter purpose, we computed a different variable for each of the two models: (i) for model 1, the threshold for stratification was set as the mean income of people in FS (i.e., 477 €/CU); (ii) for model 2, the threshold for stratification was set as the mean income of people experiencing VLFS (i.e., 160 €/CU).

Depressive mood was defined as a positive answer to the following question: “During the last 12 months, did you feel constantly sad, depressed or without any hope for at least 2 weeks?”

Living conditions were described by the type of housing (social hostels; emergency housing centers; centers for asylum seekers, or social rehabilitation centers), the number of accommodation moves since the beginning of the most recent episode of homelessness (mentioned as “number of moves” in the rest of the paper), and the housing condition before homelessness. The latter concerned the place of housing before the first experience of homelessness (mentioned as “housing conditions before homelessness” in the rest of the paper): personal or family home; housed by someone (friend, family or relative); in a shelter, a hotel or a slum; or just arrived in France. In multivariate analysis, we built a dichotomous variable “just arrived in France” versus all the other conditions in order to examine this specific population who experienced homelessness as soon as they arrived in France.

Matrimonial status (married; divorced; widowed; single; or other) and the number of children in the HH (1 or 2; 3 or more) allowed us to characterize the family situation at the time of the survey.

Social isolation was based on five questions about the frequency of interactions (by phone, email, or letters) with family members or relatives over the last 12 months, leading to a dichotomized variable (≥1 contact vs. none).

### 2.5. Statistical Analyses

Sampling weights (defined as the inverse of the inclusion probability) were computed for all participants and used for all the prevalence estimates. Chi-square tests were used to study the factors associated with food insecurity. Robust Poisson regression models were computed in order to estimate adjusted Incidence Rate Ratios (aIRR) and their 95 percent confidence intervals (CIs) in both models 1 and 2 [31]. All the covariates listed above were entered into the models and then selected with a stepwise procedure. Since there was a strong interaction between food security status, income, and some other factors (such as a depressive mood or the time spent in the current accommodation), both models were stratified by income and adjusted by 50 € increment income. In order to compare models in both strata, every covariate selected in the model of one stratum was kept in the model of the other one, whatever its significance. Statistics were performed on Stata 12^®^ (StataCorp., College Station, TX, USA).

## 3. Results

For the current analysis, we excluded the families who did not answer the HFSSM (*n* = 29, 3.6%), which led to a sample of 772 homeless families. These excluded families showed no statistical difference in their mean age or their mean income from the included ones.

### 3.1. Description of the Studied Population

Demographics, homeless duration, and number of moves in the last 12 months are summarized in Table 1.

As mentioned above, the parent interrogated in 95.4% of the cases was the mother. They were relatively young with a mean age of 32.4 years (SD = 0.33) and more than 85% were less than 40 years old; 41.1% of the respondents were married and 37.1% were single. In our sample, 21.5% of the households had three or more children. Families’ monthly income was on average 307 €/CU (SD = 16.42).

Only 6.3% of the respondents were born in France (95% CI = (4.1–9.6)); 45.0% had a nationality from a Sub-Saharan African country and 18.6% from one of the Commonwealth of Independent States.

Regarding their homeless situation, approximately 30% had been homeless since less than a year but almost 50% had been homeless for two years or more; 74.0% had experienced two moves or less in the last 12 months while almost 8% had experienced six moves or more over the same period.

### 3.2. Prevalence of Food Insecurity

Prevalence of food insecurity was at a very high level. Only 14.0% of people were FS, whereas 32.9% were in MFS, 43.3% in LFS, and 9.8% in VLFS.

### 3.3. Prevalence of FS and VLFS in Different Sub-Groups of Homeless Families 

The prevalence of FS and VLFS differed according to the type of housing. FS prevalence was higher in emergency housing centers and social rehabilitation centers (respectively 23.8% and 22.5%) while VLFS was particularly high in social hostels (11.0%, *p* = 0.04) (Table 2).

FS prevalence did not differ significantly according to the housing conditions before homelessness but the low level of FS (9.5%) in migrants who experienced homelessness for the first time upon arrival in France is worth noting. The prevalence of FS increased with the duration of the stay in the current accommodation, contrary to the prevalence of VLFS. The number of moves per year was lower in FS households (1.80 moves per year, SD = 0.18) as compared to VLFS ones (3.43 moves per year, SD = 0.34, *p* < 0.001).

VLFS prevalence was higher in people reporting a depressive mood (13.2% vs. 3.2%, *p* < 0.001 of those who did not) and FS prevalence was lower (respectively: 10.0% vs. 23.5%, *p* < 0.001).

Family composition was not statistically associated with FS status even though married people seemed to experience less frequently VLFS and families with four or more children experienced less frequently FS.

The mean income/CU/month was dramatically higher in FS families (477.8 €, SD = 34.75) than in VLFS ones (160.7 €, SD = 24.94; *p* < 0.0001).

### 3.4. Model 1: Factors Associated with Food Security in Multivariate Analysis 

In both strata, income was positively associated with FS and respondents not experiencing depressive mood were twice as likely to be food secure (Table 3).

In the first strata only people (households with an income ≤477 €/CU) that had spent more than 15 months in the current accommodation were more likely to be food secure. Also, migrant families who were already living in France when they experienced homelessness for the first time tended to be more often FS (even if the *p* value was at the border of the significance) while those living in social hostels were half as likely to be FS (aIRR = 0.42, 95% CI = (0.24–0.73)).

In the second strata—i.e., in people with a little more financial resources—housing status continued to play a role in food security (with some differences regarding these types). Families with a monthly income above 477 €/CU were more likely to be with FS if they were housed in social hostels (aIRR = 4.45; 95% CI = (1.49–13.48)) or in social rehabilitation centers (aIRR = 4.91; 95% CI = (1.69–14.29)) compared to those who lived in a center for asylum seekers. In this strata also, parents in a couple were 1.57 (95% CI = (1.02–2.41)) more likely to be food secure than those who were single, divorced, or widowed.

### 3.5. Factors Associated with Very Low Food Security in Multivariate Analysis 

In both strata, the number of moves per year and the absence of any contact with family members or relatives in the last year were both significantly associated with VLFS (Table 4).

In the first strata only the number of children (households with a monthly income ≤160 €/CU), was strongly associated with a VLFS status; a highest risk being observed among families with three children and more (aIRR = 2.58; 95% CI = (1.46–4.54)). A parental depression mood was also strongly associated with VLFS (aIRR = 3.02, 95% CI = (1.08–8.43)).

In the second strata, the housing condition before homelessness was also strongly associated with VLFS: globally, all the other situations were at higher risk of being VLFS than having experienced homelessness upon arrival in France (aIRR = 3.60; 95% CI = (1.56–8.30)).

## 4. Discussion

This study provided original insights into the food insecurity issue and associated factors in this vulnerable and understudied population. Our results showed that the homeless population in Paris urban area, characterized by foreign origins and specific familial situations (i.e., single female families, large families), was highly food insecure. Compared to the prevalence of food insecurity evaluated in 2010 in the general population living in the same urban area [22], the difference is striking (6.30% vs. 86.0%). Whereas this level of prevalence has already been observed in other studies focusing on homelessness [32,33,34,35], it is surprising and even worrying in France, a country that can be considered as a welfare state, ranking first among OECD countries for the proportion of its Gross domestic product (GDP) dedicated to public social expenditures.

Monthly income and the absence of depressive mood were both positively associated with FS. The poorest homeless families were also more likely to be with FS if they had benefited from some stability in their accommodation and if they had been housed in shelters rather than in social hostels. In homeless families with slightly higher income, asylum centers were the type of accommodation with lower FS prevalence and being in a couple was associated with a food security status.

Studying food insecurity in homeless populations allowed us to analyze thoroughly how very vulnerable families could confront signs of food security or, on the contrary, could fall into the most deleterious food insecurity. VLFS is indeed a state of disrupted eating patterns, reduced food intake, and skipped meals that affect children as well. This situation is very extreme since it is known that food insecure parents are struggling and do their best to protect their child from FI [36]. The factors leading to such an extreme situation (having had many relocation moves, being socially isolated, suffering from depression, having more than three children) were therefore particularly interesting to investigate. A study highlighted the link between housing instability and food security [6] and noted that moves require a lot of personal investment and energy for the homeless and impact their usual food access process. In our population, the families experienced a mean of 4.3 moves since they were homeless [3] and there is no doubt that such a residential instability is a source of difficulties for these families to know and access the local cheap food supply.

We observed—for the first time in France—that depression is linked with food insecurity in a homeless population. A large number of studies investigated this complex association among vulnerable families [37,38,39,40]. Depressive mood plays an important part in the way families can cope with a deprived situation and in their food security status. Conversely, the chronic stress associated with living in poverty has repeatedly been shown to have detrimental effects on mental health through various potential pathways [41,42,43].

Family composition is also an important determinant of food insecurity. We found that living as a couple was linked with FS among the wealthiest while, at the same time, the increasing number of children among the poorest subjects was associated with an increasing risk of falling into VLFS. It could easily be assumed that having two adults in the family increases and facilitates food access and that having more children increases the difficulties in providing enough food for everyone in the household.

The association between the absence of contact with family and relatives and VLFS could indicate also that the social and psychological supports usually provided by these contacts protect from the last severe form of food insecurity. Complete social and family isolation may also be the result of (or the proxy for) very traumatic personal histories (directly—e.g., domestic violence—and/or indirectly, withdrawal being a consequence of posttraumatic psychological distress [44]).

We also observed that the type of housing was linked with the food security status: homeless families in emergency housing centers and social rehabilitation centers were both more food secure than those in social hostels or centers for asylum-seekers. We searched for a potential impact of cooking facilities available in these different types of housing since we knew that, for example, fridges, cookers, and microwaves are less available in social hostels. Unfortunately, we did not find any such significant association but the available data on cooking facilities were pretty rudimentary. The quality and the importance of the social services available vary depending on the different housing structures but that cannot explain the differences observed since they are generally poorer in social hostels and also in emergency centers but better in social rehabilitation centers and even more so in centers for asylum-seekers. The hypothesis of the role of the geographical environment of the various housing structures deserves also to be raised. Indeed, some studies showed the impact of the social and local environment such as the living location, and the socioeconomic, social, and food environment on food insecurity [45,46]. The questions of the impact of social and food environment may be important in the Paris region since homeless families are increasingly housed in structures (particularly in social hostels) located far from city centers or in neighborhoods with poor public transportation services but we had no data to further explore these issues.

Finally, we found some contradictory results regarding the association between FS and housing conditions before the first experience of homelessness: families who have become homeless upon arrival in France were at higher risk of food insecurity but they were also those with a lower risk of falling into VLFS. It is not surprising that the former are at risk of FI if they were also those with higher risks of not knowing how to proceed in order to acquire some cheap or free food (through food banks, soup kitchens, or social groceries). Regarding the second result, we can assume that people with harsh living conditions at their arrival in France have, then, better knowledge or resources that prevent them falling into the most severe form of food insecurity, i.e., to protect their children from FI.

Our study has some limitations. ENFAMS study is a cross-sectional survey and this makes it difficult to be sure of the direction of some associations we observed. If food insecurity is not considered to be the cause of moving (moves are forced in accordance with the availability of accommodations), parental separation or birth of a third child, depression, and severe food insecurity may be linked in both directions as discussed above. Also our sample size was quite small and our analysis stratified, which may have led to some lack of statistical power that could explain why some factors did not remain statistically associated in multivariate analysis.

On the contrary, the ENFAMS study has some unique strength. Based on a probability sample of homeless families, it interviewed people in 17 languages allowing the cultural diversity of this population group to be taken into account. Also, we used a validated questionnaire to assess food insecurity.

## 5. Conclusions

Severe forms of food insecurity are frequent among homeless families in the Paris region, for one of the 10 wealthiest European regions and the capital region of a country that devotes a considerable part of its public budget to social welfare. From a political perspective, it seems doubtful that the situation would be the same if these homeless families were not mostly migrants. For the many social programs and professionals who work with these families, our results may help them to prevent these situations among those most at risk, i.e., those cumulating conditions such as residential instability, single parenthood, having three or more children, depressive symptoms, difficult access to cheap or free food locally, and living in social hostels are risk factors of food insecurity. Our results emphasize the need for specific public policies in order to prevent some of these risk factors. Food aid must be a top priority in this population; it is not out of reach if we consider that approximately 10,000 families were homeless at the time of the survey, accounting for 35,000 people in a region of almost 7 million inhabitants.

## Figures and Tables

**Table 1:** Characteristics of the sample.

	*n*	Weighted Proportions
Age group		
17–25	121	15.7
26–30	218	27.3
31–39	321	42.2
≥40	112	14.9
Gender		
Men	36	4.0
Women	736	96.0
Matrimonial status		
Married	302	41.1
Divorced	48	5.6
Widowed	10	1.0
Single	307	37.1
Other	105	15.3
Number of children in the HH		
1–2	587	78.4
3	130	15.5
≥4	55	6.0
Country of birth		
France	40	6.3
Abroad	732	93.7
Nationality		
Sub-Saharan Africa	346	40.6
Commonwealth of Independent States	149	16.8
Maghreb	58	10.2
Europe	61	9.6
Non Sub-Saharan Africa	53	6.8
Asia	30	4.2
Americas	10	2.0
Middle East	3	0.1
unknown	62	9.7
Homeless duration (years)		
<1	217	29.6
1–2	204	22.0
2–4	211	27.6
≥4	140	20.7
Number of moves in the last 12 months		
0	315	39.8
1–2	257	34.2
3–5	130	18.2
≥6	70	7.9

**Table 2:** Prevalence of food security (FS) and very low food security (VLFS) in different subgroups of homeless families.

	*n*	FS Prevalence (%)	95% CI	*p*	VLFS Prevalence (%)	95% CI	*p*
Type of housing							
Emergency housing centers	83	23.8	(14.3–36.9)	0.02	10.9	(5.1–21.9)	0.04
Social rehabilitation centers	103	22.5	(13.5–35.1)		3.9	(1.8–8.3)	
Centers for asylum-seekers	133	15.4	(10.1–22.9)		8.3	(4.3–15.1)	
Social hostels	453	11.7	(8.7–15.5)		11.0	(7.8–15.2)	
Housing condition before homelessness							
At his home (or family home)	95	21.3	(13.0–32.7)	0.08	3.1	(1.1–8.3)	0.15
Housed by someone	356	14.6	(10.4–20.0)		11.8	(7.7–17.5)	
In a shelter, a hotel, a slum	42	13.6	(5.6–29.4)		10.7	(4.6–22.9)	
Upon arrival in France	267	9.5	(6.0–14.6)		10.1	(6.6–37.9)	
Unknown	12	13.0	(2.7–44.0)		7.2	(0.1–15.1)	
Time spent in the present accommodation (in months, quartiles)							
<4.5	192	10.6	(6.0–18.0)	0.01	16.4	(9.6–26.6)	0.005
(4.5–7.9)	189	15.7	(9.90–24.0)		8.9	(5.3–14.5)	
(7.9–15.4)	197	7.8	(4.4–13.2)		9.9	(6.3–15.2)	
≥15.4	194	21.1	(15.4–28.1)		3.8	(1.8–7.5)	
Depressive mood							
Yes	505	10.0	(7.0–14.2)	0.001	13.2	(9.6–17.8)	<0.001
No	244	23.5	(18.0–30.2)		3.2	(1.8–5.6)	
Unknown	23	6.9	(1.8–22.6)		3.3	(0.5–18.4)	
Matrimonial status							
Married	302	15.4	(11.0–21.2)	0.056	7.6	(5.0–11.2)	0.37
Divorced	48	8.5	(4.0–17.1)		16.3	(5.9–37.9)	
Widowed	10	41.3	(12.6–77.4)		0.0	-	
Single	307	10.6	(7.7–14.5)		11.3	(7.7–16.2)	
Other	105	18.4	(10.9–29.5)		10.4	(5.4–19.1)	
Number of children in the household							
1–2	587	12.8	(9.8–16.6)	0.16	8.9	(6.0–12.8)	0.16
3	185	18.1	(12.2–26.0)		13.25	(8.6–19.9)	
Contacts with family members or relatives over the last 12 months							
≥1	692	14.1	(11.2–17.7)	0.73	9.4	(6.8–13.0)	0.14
None	80	12.4	(6.0–23.9)		15.9	(8.7–27.3)	

**Table 3:** Model 1: factors associated with food security (stratified multivariate analysis).

	Among Families with Monthly Income ≤ 477 €/CU (*n* = 559)			Among Families with Monthly Income > 477 €/CU (*n* = 159)		
	aIRR	95% CI	*p*	aIRR	95% CI	*p*
Monthly income per consumption unit /50 €	1.16	(1.07–1.25)	<0.001	1.04	(1.01–1.07)	0.008
Depressive mood						
Yes	Ref.	-	-	Ref.	-	-
No	2.37	(1.50–3.73)	<0.001	2.32	(1.51–3.58)	<0.001
Time spent in the current accommodation (in months)						
<15	Ref.	-	-	Ref.	-	-
≥15	1.75	(1.09–2.8)	0.02	1.19	(0.77–1.83)	0.438
Type of housing						
Centers for asylum-seekers	Ref.	-		Ref.	-	-
Social hostels	0.42	(0.24–0.73)	0.002	4.45	(1.47–13.48)	0.008
Emergency housing centers	0.96	(0.49–1.88)	0.91	3.74	(0.97–14.39)	0.055
Social rehabilitation centers	1.17	(0.57–2.38)	0.67	4.91	(1.69–14.29)	0.004
Housing condition before homelessness						
Upon arrival in France	Ref.	-	-	Ref.	-	-
Other	1.69	(0.99–2.9)	0.054	0.94	(0.58–1.52)	0.788
Matrimonial status						
Single, divorced, widowed	Ref.	-	-	Ref.	-	-
In a couple	1.10	(0.68–1.78)	0.699	1.57	(1.02–2.41)	0.039

**Table 4:** Model 2: factors associated with very low food security (stratified multivariate analysis).

	Among Families with Monthly Income ≤ 160 €/CU (*n* = 116)			Among Families with Monthly Income > 160 €/CU (*n* = 133)		
	aIRR	95% CI	*p*	aIRR	95% CI	*p*
Monthly income per consumption unit/50€	0.90	(0.66–1.24)	0.53	1.00	(0.94–1.07)	0.93
Number of moves per year	1.08	(1.03–1.14)	0.004	1.18	(1.08–1.29)	<0.001
Number of children in the household						
1 or 2	Ref.	-	-	Ref.	-	-
3 and more	2.58	(1.46–4.54)	0.001	0.42	(0.1–1.81)	0.24
Depressive mood						
No	Ref.	-	-	Ref.	-	-
Yes	3.02	(1.08–8.43)	0.03	0.69	(0.3–1.55)	0.37
Contacts with family members or relatives over the last 12 months						
≥1	Ref.	-	-	Ref.	-	-
None	1.96	(1.06–3.62)	0.03	2.36	(1.12–4.97)	0.02
Housing condition before homelessness						
Upon arrival in France	Ref.	-	-	Ref.	-	-
Other	0.90	(0.66–1.24)	0.53	3.60	(1.56–8.30)	0.003
